# Transcriptome analysis of neo-tetraploid rice reveals specific differential gene expressions associated with fertility and heterosis

**DOI:** 10.1038/srep40139

**Published:** 2017-01-10

**Authors:** Haibin Guo, Jean Nestor Mendrikahy, Lei Xie, Junfeng Deng, Zijun Lu, Jinwen Wu, Xiang Li, Muhammad Qasim Shahid, Xiangdong Liu

**Affiliations:** 1 State Key Laboratory for Conservation and Utilization of Subtropical Agro-bioresources, South China Agricultural University, Guangzhou 510642, China

## Abstract

Polyploid rice hybrids have a powerful biological and yield potential that may become a new way for rice breeding; however, low fertility is major hindrance in commercial utilization. Here, we developed a neo-tetraploid rice that could overcome the sterility of autotetraploid rice and produce high heterosis. Transcriptome analysis of F_1_ hybrid developed by crossing neo-tetraploid with autotetraploid rice displayed 807, 663 and 866 differentially expressed genes that uniquely associated with F_1_ and specific to (DEGFu-sp) anther, ovary and leaf, respectively. Of the DEGFu-sp, 1224 genes displayed nonadditive expression; 44 and 10 genes were annotated as TFs and methyltransferase or hydroxymethyltransferase, respectively. Gene ontology enrichment and co-expression analysis revealed specific differential gene expressions in the DEGFu-sp to leaf, anther and ovary, such as genes related to photosynthesis, metabolic process and transport, and co-expression network including fertility, resistance and epigenetic elements. Of the DEGFu-sp to anther, 42 meiosis stage-specific genes, eight meiosis-related genes, such as *RAD51* and *SMC2*, were identified. We identified 38 miRNAs from DEGFu-sp to anther, and their targets were associated with pollen fertility and retrotransposon protein. Our study provides new germplasm for polyploid rice breeding, and revealed complex regulatory mechanisms that might be associated with heterosis and fertility.

Rice is one of the main food crops of the world’s population. Heterosis or hybrid vigor is a phenomenon which refers to the superior performance of a hybrid relative to one or both parental phenotypes. Heterosis had been significantly increased the yield of rice crop in the world during last fifty years^1^. However, the rice yield is stagnant from the last few years. So, it is of utmost importance for the rice breeders to breed new rice varieties with high yield and resistant against various biotic and abiotic stresses. Rice polyploidization can improve the heterozygosity of “allogeneic”, and increases the probability of combination and interaction of elite genes, thereby, increases the adaptability to adverse environmental conditions^2^,^3^,^4^,^5^.

Polyploidy plant does not only provide a large number of material basis for mutants, evolutionary adaptation and selection, but also has become one of the important ways to promote evolution and plant breeding^6^. Intersubspecific hybrids (*indica* × *japonica*) of autotetraploid rice have a powerful biological and yield potential, and it is expected to become a new way to breed rice in the future. Therefore, polyploidy rice has attracted the attention of rice scientist^3^,^4^,^5^,^7^,^8^,^9^.

However, autotetraploid rice has many unfavorable traits, just like other newly synthesized polyploids. Among these unfavorable traits, low fertility has the largest negative impact on rice breeding, and it is difficult to take out the advantages of gigantic features of autotetraploid rice, so autotetraploid rice hardly applied in commercial production^10^,^11^. Recently, we revealed that polyploidy strengthen F_1_ pollen sterility multi-loci interactions, which cause meiosis abnormalities and resulted in high pollen sterility in autotetraploid rice hybrids^10^. Consequently, how to create new tetraploid rice lines with normal fertility and to overcome the sterility of F_1_ hybrids is a key step to use the tetraploid rice. To solve this problem, after 20 years of unremitting efforts, our research group has successfully bred a number of new tetraploid rice lines with high seed setting (>80%). Our research group has registered two new lines of tetraploid rice for PVP (Protection for New Varieties of Plants) in China^4^. These new tetraploid rice lines with high fertility were designated as neo-tetraploid rice according to neo-tetraploid *Arabidopsis*^6^.

RNA-seq-based transcriptome data provide very helpful platform for investigating the molecular aspects of heterosis in plants. The complexity of gene expression profiles associated with heterosis and differential gene expressions in different tissues had been revealed using transcriptome in rice^12^,^13^, maize^14^,^15^ and wheat^16^. Therefore, we concentrated on the transcriptomic data to analyze the molecular aspects of heterosis and fertility of neo-tetraploid rice in the present study. We reported the breeding procedure of Huaduo 3, a neo-tetraploid rice line, and heterosis and transcriptomic analysis of hybrids developed by crossing Huaduo 3 with different autotetraploid rice lines. Our study provides new germplasm for polyploid rice breeding and reveals some genes that may associate with the fertility and heterosis in neo-tetraploid rice.

## Results

### The breeding procedure of neo-tetraploid rice, Huaduo 3

**Figure 1:**
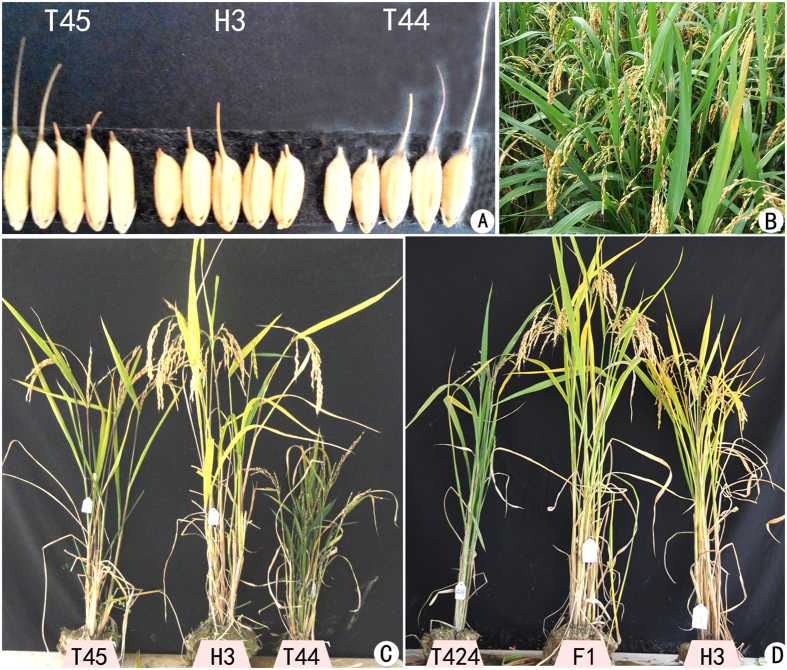
Morphological characteristics of F_1_ hybrid and their parents. (**A**) Grains of Jackson-4x (T45), Huaduo 3 (H3) and 96025 (T44). H3 was developed from T45 and T44; (**B**) Huaduo 3 at grain filling stage in a paddy field; (**C**) Plant appearance of 96025 (T44), Huaduo 3 (H3), and Jackson-4x (T45); (**D**) Plant structure of Shennong15-4x (T424), Huaduo 3 and their F_1_ hybrid.

In this study, an autotetraploid rice line, Jackson-4x, and another autotetraploid rice line, 96025, were self-crossed for more than 10 years at our farm. The combination of Jackson-4x ×96025 was generated in 2005. The F_1_ hybrid was harvested and continuously self-crossed until F_4_, and one plant with more than 80% seed setting was found in F_5_. We planted more than 60 plants with high fertility from the F_5_ plant, and continuously planted in the field from F_7_ to F_11_. These lines maintained high seed setting in next successive generations (F_10_ to F_13_), and named as “Huaduo 3” in 2012 (Supplementary Fig. S1). Huaduo 3 (H3) exhibited “high fertility”, and has an excellent plant type and yield-related traits (Fig. 1). Cytogenetic observations revealed more than 90% normal pollen mother cells in meiosis (Supplementary Fig. S2).

### Heterosis analysis of neo-tetraploid rice

The agronomic traits of 33 control autotetraploid hybrids and 40 other hybrids generated by crossing with various autotetraploid lines (i.e., H3 crossed with 26 *indica* and 14 *japonica* autotetraploid rice lines) improved significantly compared to their parents, especially the average values of the traits like filled grains per plant, seed setting and grain yield per plant for the hybrids prepared by H3. Of these traits, filled grains per plant were more than 662, the seed setting was more than 76%, and grain yield per plant was more than 22 g for all hybrids. The average values of filled grains per plant, seed setting and grain yield per plant for the hybrids were in the following order: hybrids of H3 and *indica* autotetraploid lines > hybrids of H3 and *japonica* autotetraploid lines > hybrids of *indica* and *japonica* autotetraploid lines (Supplementary Table S1). It is worth mentioning that the seed setting of the hybrids from H3 was significantly higher than those hybrids generated from other autotetraploid lines.

For the 26 hybrid combinations between H3 and *indica*, the mid-parent heterosis values were positive for all the traits except width of ten grains. A total of 15 hybrids showed positive high-parent heterosis for six traits, except length and width of 10-grains (Supplementary Fig. S3, Table S2). For 14 combinations generated by crossing H3 with *japonica* autotetraploid lines, the average value of high-parent heterosis for five agronomic traits, including plant height, effective number of panicles per plant, filled grains per plant, total grains per plant and grain yield per plant, were positive. The highest high-parent heterosis was detected for the total grains per plant (i.e. 68.12%), followed by the effective number of panicles per plant > filled grains per plant > grain yield per plant > plant height (Supplementary Fig. S3, Table S3).

For further analysis, we selected one hybrid, Huajingxian 74-4x (T452) ×H3, to analyze F_1_ heterosis and to investigate the transcriptome of hybrid and its parents. T452 is an autotetraploid rice line with low fertility. The hybrid showed positive high-parent heterosis for all the traits except grain length and width of 10-grains, and the values for high parent heterosis were very high for effective number of panicles per plant and grain yield per plant (Table 1). All the traits showed positive mid-parent heterosis values except for the width of 10-grains.

### Whole genome transcriptome profiles of F_1_ and its two parental lines are of satisfactory quality

Since we found high yield-related traits heterosis in F_1_ compared to its parents, and higher F_1_ fertility than T452, so the transcriptome profiles of F_1_ and its two parental lines were analyzed in three tissues, including anthers and ovaries at meiosis stage, and flag leaves during flowering. In total, we detected more than 548 million clean reads from the F_1_ and its parents by sequencing the transcriptomes of three tissues. By aligning the reads against the Nipponbare reference genome, an average of 89.06% annotated transcripts of the reference genome was obtained in our material, which was much greater than the minimum value (70%). The average percentage of unique mapped reads was 93.45%. There was a significant correlation between three biological replicates of transcriptome data in hybrid and its parents, and correlation coefficient was more than 0.8. The qRT-PCR was employed to validate the transcriptomic data by using 12 primers and the results showed that the expression profiles of 12 differentially expressed genes (DEGs) were consistent with the transcriptome data (Supplementary Fig. S4).

**Figure 2:**
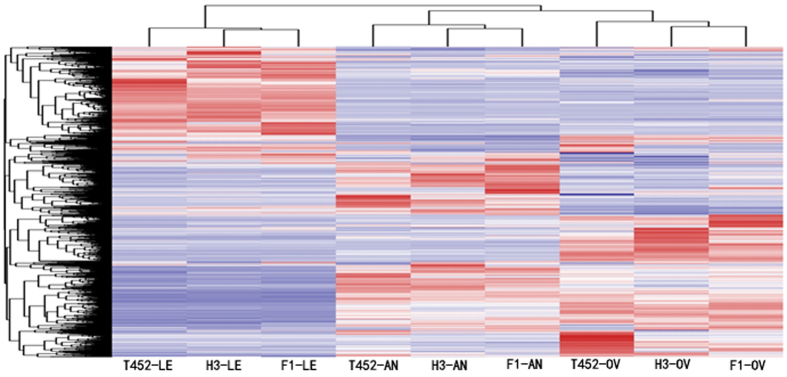
Hierarchical clustering analysis of all expressed genes based on transcriptome data. T452: Huajingxian 74-4x; H3: Huaduo 3; F_1_: (Huajingxian 74-4x ×Huaduo 3); AN: Anther; OV: Ovary; LE: Leaf.

Hierarchical Cluster analysis was employed to investigate the correlations between differentially expressed genes in F_1_ and its two parents using transcriptomic data at different stages, and the results revealed that the three tissues, anther, ovary and leaf, from the F_1_ and its parents always assembled into primary groups at the same stage (Fig. 2). Moreover, we also found that most of the expressed genes in F_1_ were more similar to those in H3 (Supplementary Fig. S5), suggesting that the transcriptome profiles were consistent with the phenotypic data of F_1_ and its two parents.

### Identification of differentially expressed genes (DEGs) and functional annotation of DEGs associated with anther, ovary and leaf in F_1_ and its parents

A total of 17877 DEGs were detected in three tissues, anther, ovary and leaf, of F_1_ and its parents, and ranged from 1521 to 2498 DEGs between F_1_ compared to its parents and between two parents (Table 2). DEGs between the hybrid and its parents are designated as DEGF, and the DEGs between the parents are called as DEGP. The DEGF could be divided into two groups, i.e. one shared by DEGP and DEGF, and another uniquely belonging to F_1_ compared to parents, which was designated as DEGFu. The DEGFu could explain the phenotypic differences between F_1_ and its two parents^12^; therefore, we specifically focused on the DEGFu to find out the genes associated with the heterosis and fertility. Of the 17877 DEGs, we obtained 1150, 1014 and 1122 DEGFu associated with anther, ovary and leaf between F_1_ and its parents, respectively. Among DEGFu, 807, 663 and 866 were specifically associated with anther, ovary and leaf, respectively (hereafter referred as DEGFu-sp). Among the 2336 DEGFu-sp, 10 genes encoding methyltransferase or hydroxymethyltransferase were detected (Supplementary Table S4).

To understand the functions of DEGFu-sp genes in different tissues, we investigated the transcription factors (TFs), and found that 44 TFs, including 16 in anther, 10 in ovary and 18 in leaf, exhibited different levels of expressions in F_1_ compared to its parents. Meanwhile, we also used DEGFu-sp genes to identify protein-protein interactions, and the results revealed significant interactions in different tissues. Eight subnetworks, and 14 significant pathways, such as metabolic pathways, starch and sucrose metabolism, were detected in anther. A total of 17 subnetworks, and three significant pathways, such as Diterpenoid biosynthesis and biosynthesis of secondary metabolites, were identified in ovary. Moreover, we detected seven subnetworks and 32 significant pathways related to DEGFu-sp genes in leaf (Supplementary Fig. S6a–c and Table S4).

Then, gene ontology (GO) enrichment analysis was employed for the functional categorization of genes, and the results revealed that differential gene expressions might be associated with fertility and heterosis in anther, ovary and leaf. We identified 19 prominent categories associated with DEGFu-sp to anther in biological process category that were mainly related to photosynthesis, regulation of metabolic process, regulation of biosynthetic process and regulation of transcription (Supplementary Fig. S7). Six pathways were involved in the DEGFu-sp to anther, such as photosynthesis and metabolic pathways. Here, 13 genes showed high levels of expressions in F_1_ compared to T452. Of 807 DEGFu-sp to anther, 15 genes exhibited co-expression with 46 other important genes (Supplementary Table S4).

The DEGFu-sp to ovary could be divided into 38 clusters, which showed 13 prominent functional gene groups that associated with metabolic and biosynthetic processes in the biological process category, and microtubule associated complex (Supplementary Fig. S8a,b). Among 663 DEGFu-sp to ovary, 26 genes showed co-expression with 79 other genes (Supplementary Table S4).

**Figure 3:**
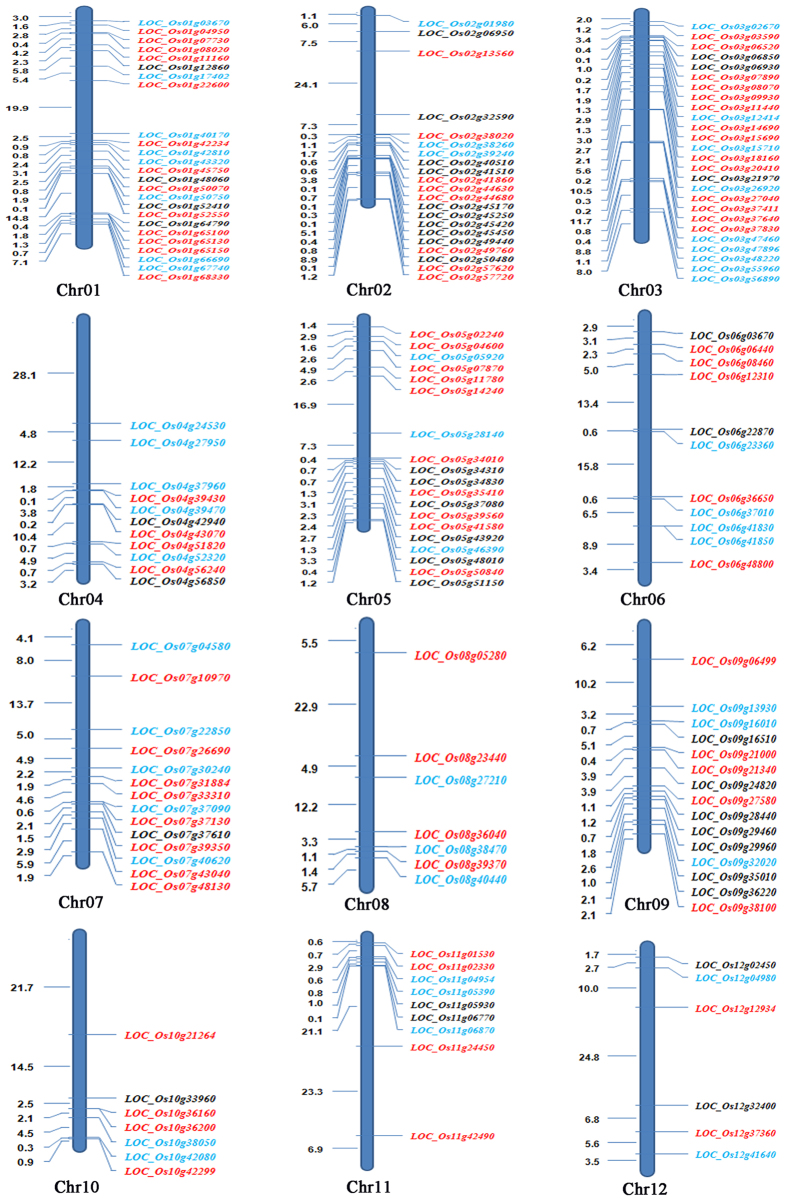
Location of genes associated with fertility and heterosis, and specific to leaf, anther and ovary. Genes in red, blue and black letters associated with transport (leaf), meiosis (anther) and metabolic process (ovary), respectively.

The 866 DEGFu-sp to leaf were mainly categorized into 62 clusters, and 32 prominent genes groups were detected, including 81 genes related to transport (Fig. 3), localization and small molecule metabolic process (Supplementary Fig. S9). We identified 87 genes associated with metabolic pathways (Supplementary Table S4), among them, five genes displayed high levels of expressions in F_1_ compared to T452. They were annotated as aldehyde dehydrogenase (*LOC_Os02g43280*), aminotransferase (*LOC_Os03g07570*), cytochrome P450 (*LOC_Os03g12660*), GHMP kinases ATP-binding protein (*LOC_Os06g48940*) and molybdenum cofactor biosynthesis protein 1 (*LOC_Os12g32230*). Since H3 is an excellent neo-tetraploid rice line with high fertility and its leaves color changed with the passage of time during flowering, and T452 is an autotetraploid rice line with low fertility and leaves stay-green during flowering. Therefore, we compared the up-regulated genes between F_1_ compared to T452 and H3 compared to T452, and 74.01% up-regulated genes were similar in both comparisons. Interestingly, we detected two main prominent functional gene classes in leaf, including programmed cell death and defense response. Among 866 DEGFu in leaf, 19 genes displayed co-expression with 20 other important genes (Supplementary Table S4).

### Differentially expressed anther specific genes were associated with meiosis stage-specific genes between F_1_ and its parents

Since we used transcriptome to analyze the DEGF in neo-tetraploid hybrid during pollen mother cell (PMC) meiosis, and T452 is an autotetraploid rice line with low fertility, we specifically investigated meiosis stage-specific genes associated with the differentially up-regulated genes in F_1_ compared to T452 to identify genes related to high fertility of F_1_. Firstly, we compared the up-regulated genes in F_1_ compared to T452 with those up-regulated genes in H3 compared to T452, and 643 specifically expressed genes were detected (Supplementary Table S5). AgriGo analysis of the 643 genes displayed six prominent functional gene groups associated with defense response, photosynthesis, cell death and programmed cell death. Among these genes, seven genes were related to photosynthesis (GO:0015979). A total of 41 genes exhibited down-regulation in autotetraploid hybrid with low fertility compared to diploid rice (Supplementary Table S5)^10^. Of these 41 genes, four genes, including *LOC_Os04g33830, LOC_Os12g08770, LOC_Os06g21590* and *LOC_Os07g25430*, were associated with photosynthesis. AgriGo analysis of the anther specific up-regulated genes revealed that there were no significant GO terms in F_1_ compared to H3.

Secondly, we compared 643 anther specific up-regulated genes in F_1_ compared to T452 with microarray data of wild type rice anther meiosis stage-specific expression^17^,^18^, and meiosis-related expression^18^,^19^,^20^. We identified nine overlapping genes that showed meiosis stage-specific expression from zygotene to tetrad (Supplementary Table S5).

Thirdly, we compared DEGFu-sp to anther with the microarray data of wild type rice meiosis stage-specific genes^17^,^18^ and meiosis-related genes^18^,^19^,^20^, and 42 meiosis stage-specific and eight meiosis-related overlapping genes were detected (Fig. 3). Of the eight meiosis-related genes, two genes, including *LOC_Os11g04954* and *LOC_Os12g04980*, encoded DNA repair (*RAD51*) family protein, and one gene, *LOC_Os01g67740*, was annotated as structural maintenance of chromosomes protein 2 (SMC2) (Supplementary Table S5). Moreover, four genes associated with photosynthesis, including *LOC_Os04g33830, LOC_Os12g08770, LOC_Os06g21590* and *LOC_Os07g25430*, exhibited down-regulation in autotetraploid hybrid with low fertility compared to diploid rice^10^.

Since transcriptomes may also be regulated through epigenetic mechanisms^21^, we also analyzed micro-RNA (miRNA). A total of 288 differentially expressed miRNAs (DERs), including 13 novel miRNAs, were detected in anther. Of these DERs, 38 were uniquely belonging to F_1_ compared to its parents. Together, 397 target genes were predicted by the 38 DERFu (Differentially expressed miRNA uniquely belonging to F_1_ compared to its parents) (Table 3 and Supplementary Table S6). Gene ontology (GO) enrichment analysis of the 397 targets showed seven prominent functional gene groups related to cell death, defense response, programmed cell death, apoptosis, DNA integration and response to stress. Most of the targets were the same as those detected by a comparison between the up-regulated genes in F_1_ compared to T452 and up-regulated genes in H3 compared to T452. Of the 397 targets, five genes were annotated as ubiquitin-conjugating enzyme or ubiquitin family protein, three genes encoded BURP-domain containing protein, four genes enriched in ATP binding protein, one gene was involved in chlorophyll a-b binding protein 1, seventy seven genes were associated with retrotransposon and nine genes encoded transposon protein or transposable element protein (Supplementary Table S6). Then we compared 38 DERs uniquely belonging to F_1_ compared to its parents with previously identified differentially expressed miRNAs. We detected one miRNA, *osa-miR5072_L-2_1ss3AG*, which was down-regulated in meiosis between Taichung65-2x and Taichung65-4x during pollen development, and predicted a target, *LOC_Os02g24960*, which was annotated as retrotransposon protein^22^.

### Nonadditive gene expression in anther, ovary and flag leaf of F_1_ hybrid

The gene expression in F_1_ could be divided into two types, the first was designated as additive expression that contributed by each allele from both parents, and another was nonadditive expression that deviates from the mid-parent value^12^. A total of 1224 nonadditive differentially expressed genes (NDEGs) were detected in the three tissues, including 314, 578 and 332 in anther, ovary and leaf, respectively (Supplementary Table S7). Of the 1224 NDEGs, 895 and 329 genes were found to be up- and down-regulated, respectively (Table 4). We compared the NDEGs in anther with the microarray data of rice anther meiosis specific genes^17^,^18^,^19^,^20^, and found 53 overlapping genes between anther and microarray data in meiosis. Among 53 genes, seven genes, including *LOC_Os07g40620, LOC_Os02g38260, LOC_Os01g65630, LOC_Os03g21090, LOC_Os05g41120, LOC_Os04g49260* and *LOC_Os05g38350*, displayed down-regulation in autotetraploid hybrid compared to diploid rice with low fertility^10^. These seven genes were annotated as putative uncharacterized protein.

The Plant GeneSet Enrichment Analysis Toolkit (PlantGSEA) was employed to analyze the gene set enrichment of 578 NDEGs of ovary, and it showed a prominent category comprising of 74 genes that associated with a primary metabolic process (GO:0044238) in biological process. Moreover, we detected a microtubule associated complex (GO:0005875) that related to four genes in cellular component. These four genes encoded kinesin motor domain containing protein. Among the 332 NDEGs of leaf, nine prominent enrichment gene groups were detected, which mainly related to response to stress (GO: 0006950), defense response (GO: 0006952), programmed cell death (GO:0012501) (Supplementary Table S7).

### Association between DNA sequence variations and differentially expressed genes (DEGs) in F_1_ compared to its parents

DNA re-sequencing revealed a huge difference in genome sequence of maternal, T452, and paternal, H3. A total of 2351039 SNPs were detected in T452 and H3, among them 1192412 and 374460 SNPs were specifically expressed in T452 and H3, respectively. A total number of 499448 Indels were observed in T452 and H3, of these 248194 and 84196 Indels showed specific variation in T452 and H3, respectively (Supplementary Table S8). The polymorphism rates of SNPs and Indels were 66.77% and 69.97% between T452 and H3, respectively (Supplementary Table S9). Of those polymorphic loci, about 65% of SNPs and 72% of Indels were in genic regions, 47.67% SNPs and 53.37% Indels were found to be in up or down regulatory regions that might be associated with the differentially expressed genes (DEGs). Moreover, we detected 58908 SNPs and 5561 Indels in coding regions (Supplementary Fig. S10 and Table S10).

Since many DNA sequences variation were detected between T452 and H3, we detected genes variation in DEGFu-sp, and a total of 330, 291 and 459 genes showed DNA sequence variation (SNP+Indel) in anther, ovary and leaf, respectively (Supplementary Table S7). The genes of three groups displayed differential expressions in anther, ovary and leaf of F_1_ compared to its parents. Of the 330 genes in anther, we detected 15 prominent categories in biological process, mainly related to photosynthesis, regulation of cellular metabolic process, regulation of transcription and biosynthetic process. We identified a prominent enrichment category associated with metabolic process (GO:0008152), consisting of 98 genes in biological process of ovary. Eight prominent enrichment genes, mainly related to cellular nitrogen compound metabolic process, establishment of localization and transport, were found in biological process category of leaf. These main enrichment categories were nearly consistent with the DEGFu-sp to anther, ovary and leaf (Supplementary Table S7).

Moreover, 67, 133 and 87 genes involved in SNP and Indel variations between two parents were detected in nonadditive differentially expressed genes (NDEGs) of anther, ovary and leaf, respectively. We detected 25 and 39 metabolic process related genes in anther and ovary, respectively. Notably, we found 25 genes related to TFs involved in SNP and Indel variations between parents (Supplementary Table S7).

## Discussion

Both hybridity and induced polyploidy are the potent sources to increase the biomass, yield and resistance against various biotic and abiotic stresses. Agronomic traits, such as larger grain size, stronger stem, and longer panicles of autotetraploid rice than their corresponding parents, are of special interest for rice breeders^2^,^8^,^9^,^10^,^11^,^23^. However, low seed set has been a major obstacle in the use of autotetraploid rice and cannot be used directly in the breeding programs^10^,^11^. Autotetraploid lines with high seed setting might be developed through artificial selection of plants generated by the crossing of *indica* and *japonica* autotetraploid rice lines^24^,^25^. Here, we reported a neo-tetraploid rice, Huaduo 3, with high fertility (>80%) that was developed in 2012 by our research group. We developed 40 tetraploid rice hybrids by crossing H3 with different autotetraploid rice lines to observe the fertility and heterosis, and found encouraging results.

Firstly, our results revealed that H3 has the ability to improve the seed setting of different autotetraploid rice lines by hybridization. For example, a hybrid between H3 and autotetraploid rice line, with a seed setting of 8.43%, exhibited a normal seed set (67.08%). Another hybrid between H3 and autotetraploid rice line with seed setting of 42.97%, yielded high levels of seed set (84.73%). Overall, all the hybrids generated by H3 showed high seed setting (>80%), though some parental lines had very low seed setting.

Secondly, we detected positive and higher levels of heterosis for the yield and yield-related traits, such as effective number of panicles per plant, filled grains per plant, total number of grains per plant and grain yield per plant in the hybrids generated by H3. These results are consistent with the previous studies who found that autotetraploid hybrids had high-parent heterosis for filled grains per panicle, seed setting and yield^7^,^8^,^9^. However, these studies were limited to few lines or/and hybrids. Together, our work strongly suggests that H3 is not only producing hybrids with high fertility, but also exhibited powerful hybrid vigor for yield and yield-related traits compared to other autotetraploid lines.

In the present study, length and width of 10-grains showed no positive high-parent heterosis. These results are in agreement with the previous studies, who also found negative high-parent heterosis for grain length and width in autotetraploid rice^8^,^9^. Guo *et al*.^25^ investigated several autotetraploid rice hybrids, and the results showed that the mid-parent heterosis for grain length and width was significant, but high-parent heterosis exhibited opposite trends, i.e., the former was negative, and the latter was positive. We inferred that a complex genetic mechanism controlled the heterosis for length and width of 10-grains in autotetraploid rice.

The complexity of gene expression profiles associated with heterosis and differential gene expressions in different tissues had been revealed using transcriptome in diploid rice^12^,^13^. For example, genes associated with energy metabolism and transport enriched in DGHP (differentially expressed genes between the hybrid and its parents) rather than in DGPP (differentially expressed genes between the parental lines), and the genes involved in starch synthesis had much higher expression in the panicle of F_1_ than sterile parent at grain filling stage^12^. A transcriptome analysis of the root heterosis showed that DGHP were significantly enriched in carbohydrate metabolism and plant hormone signal transduction^26^. RNA-seq data of two rice genotypes and their reciprocal hybrids showed that DEGs tend to have tissue-specific expression patterns^27^. Moreover, RNA-seq-based transcriptome profiling analysis has revealed that polyploidy level is greatly associated with genome-wide disruption of gene expression and ultimately altered phenotypes in the polyploid rice hybrids (*indica* × *japonica*)^28^. Transcriptome analysis revealed that about 16% of the 16,112 expressed genes showed nonadditive expressions in leaf tissue of an interspecific F_1_ triploid hybrid^29^. The differential gene expressions for cell growth and functional secondary metabolites were found in induced autotetraploid of Chinese Woad (*Isatis indigotica* Fort.) by transcriptomic analysis^30^.

In the present study, the differentially expressed genes that uniquely associated with F_1_ and specific to (DEGFu-sp) anther were mainly enriched in four pathways, including photosynthesis, regulation of metabolic process, regulation of biosynthetic process and regulation of transcription. The DEGFu-sp genes to ovary were involved in metabolic process, biosynthetic process and response to stimulus. Interestingly, a prominent functional gene group associated with microtubule complex was detected in ovary. In higher plants, microtubule associated proteins are involved in the regulation of microtubules assembly and disassembly, and also responsible for the organization of microtubule structures and functions^31^. Therefore, we speculate that DEGFu-sp to microtubule-associated complex may involve in the embryo sac fertility.

Most of the leaf DEGFu-sp genes expressed in F_1_ were similar to that in Huaduo 3, and consisted of significantly enriched genes in transport, localization, and small molecule metabolic process. We also detected genes related to metabolic pathways. Moreover, more than 70% up-regulated genes in F_1_ were also found to be up-regulated in H3 compared to T452. The genes associated with leaf displayed two main prominent functional gene classes, including programmed cell death and defense response. These genes may be associated with energy transfer and heterosis, which inherited from the neo-tetraploid rice, Huaduo 3.

The dosage effect of transcription factors (TFs) had been considered as an important factor to affect phenotypes of hybrids in rice^12^. In the present study, we obtained 44 TFs that showed different expressions in DEGFu-sp, and the number of TFs was less than those (187 TFs) detected in diploid rice hybrids. Since previous study had revealed that DNA methylation and activity of class II transposable elements (TEs) involved in gene expression in autotetraploid rice^32^, this may be associated with the decrease in the number of TFs in F_1_ compared to its parents in our study. Meanwhile, we detected 25 TFs involved in SNP and Indel variations between the parents, T452 and H3. These TFs may alter the gene expression and is a possible molecular mechanism underlying heterosis and fertility in neo-tetraploid rice^12^. Among DEGFu-sp, we found 10 genes annotated as methyltransferase or hydroxymethyltransferase, which were related to epigenetic genes. Moreover, about 3% of total genes displayed nonadditive expressions, which might be regulated by *trans*-acting factors^33^. These results are consistent with the previous studies, who also found nonadditive genes in rice^12^,^14^,^29^. All of these results demonstrated complex regulatory mechanisms associated with heterosis, and differential gene expression was involved in fertility of neo-tetraploid rice hybrids.

Meiosis is a conserved process associated with many key genes in rice reproduction. More than 400 meiosis-specific or stage-specific genes have been identified in rice anther, and 28 genes were characterized in rice meiosis^17^,^18^,^19^,^20^,^34^. Since there are four chromosomes in autotetraploid rice, meiosis played more crucial role in fertility. A total of 55 meiosis-related or meiosis-stage-specific genes involved in pollen sterility loci interactions in autotetraploid rice hybrids were detected in our previous study^10^. In the present study, 42 meiosis stage-specific and eight meiosis-related genes revealed differential expressions in DEGFu-sp to anther. Of the 42 meiosis stage-specific genes, 26 genes were found to be down-regulated in autotetraploid hybrid with low fertility, and most of them were key genes for pollen fertility, such as *Os09g0493500* that encoded ubiquitin fusion degradation protein and *Os09g0329000* that was annotated as BURP domain containing protein^10^. These results suggested that fertility could be improved by suppressing alternation of the expression profiles of important meiosis-related genes, and by reducing epistatic interactions between alleles of pollen sterility loci in neo-tetraploid rice.

miRNA, an important epigenetic factor, regulates the changes in transcriptomes associated with heterosis and fertility^21^,^22^. Here, 38 miRNAs, uniquely belonging to F_1_ compared to its parents, were identified, and they predicted 397 target genes. Of these targets, 13 targets were found to be involved in pollen fertility, and 86 target genes were related to retrotransposon or transposon protein or transposable element protein. Moreover, one miRNA, *osa-miR5072_L-2_1ss3AG*, which predicted a target, *LOC_Os02g24960*, and annotated as retrotransposon protein, was detected in anther meiosis. Since DNA methylation variation of transposable elements are widespread and had a remarkable effect on gene expression in autotetraploid rice^22^,^32^, the changes in rich retrotransposon, transposon and transposable elements may act as a “super-genome shock” response factor for creating adaptability and to produce high fertility and heterosis in neo-tetraploid hybrids.

### Plant Material

A neo-tetraploid rice, Huaduo (H3), and 40 different autotetraploid rice lines were used for developing hybrids in the present study (Supplementary Table S11). A total of 73 hybrids were developed, including 33 hybrids (control, CK) were generated by crossing different autotetraploid rice lines with each other (Supplementary Table S12), and 40 hybrids were developed by crossing H3 with different autotetraploid rice lines (Supplementary Table S13).

## Methods

### Evaluation of agronomic traits and data analysis

A total of 15 plants of each parent and F_1_ hybrid were harvested from the field at maturity. Agronomic traits, including plant height, effective number of panicles per plant, filled grains per plant, empty grains per plant, total grains per plant, seed setting ratio, grain yield per plant, length and width of ten grains of the selected plants, were measured. These agronomic traits were selected and investigated or measured according to the protocols of People’s Republic of China for the registration of a new plant variety DUS (Distinctness, Uniformity and Stability) test guidelines of rice (*Oryza sativa* L) (Guidelines for the DUS test in China, 2012)^4^.

The single factor variance analysis of each trait (different combinations) was done by SPSS 16.0. Multiple comparison was done by Duncan’s New Multiple-Range test (DMRT), using α = 0.05 significant level. High-parent heterosis (HPH) and mid-parent heterosis (MPH) were estimated by the following formula: HPH = (F_1_ − HP)/HP × 100%, and MPH = (F_1_ − MP)/MP × 100%, where F_1_ is the performance of first filial generation (hybrid), HP is the performance of the best parent, and MP is the average performance of two parents.

### Whole-genome re-sequencing analysis

Young Leaves of Huaduo 3 and Huajingxian 74-4x were collected and stored at −80 °C for DNA isolation. Genomic DNA (gDNA) was extracted from each young leaf tissue by using a modified CTAB method^35^. The task of whole-genome re-sequencing was performed on an Illumina HiSeq2000™ by Biomarker Technologies (Beijing, China). The procedure was performed in accordance with the standard Illumina protocol as described previously^36^. The detection of SNPs and Indels were mainly performed by GATK software tools (https://www.broadinstitute.org/gatk/guide/best-practices.php). The SNPs and Indels annotations were performed based on the reference genome data by using SnpEff software.

### Transcriptome analysis

Anthers and ovaries at meiosis stage, and flag leaves during flowering were collected from Huaduo 3, Huajingxian 74-4x and their F_1_ hybrid. All samples were collected in three biological replicates and stored at −80 °C for RNA isolation. The total RNA from each sample was extracted from the anthers, ovaries and leaves according to the manual instruction of the TRlzol Reagent (Life technologies, California, USA). The samples from anther and ovary were mixed from three biological replicates for RNA extraction. The quantity and quality of each RNA sample were assessed using 1% agarose gel and examined with a Nanodrop 1000 spectrophotometer (Nanodrop, Wilmington, DE, USA). RNA integrity number and concentration were checked using an Agilent 2100 Bioanalyzer (Agilent Technologies, Inc., Santa Clara, CA, USA). The mRNA was isolated by NEBNext Poly (A) mRNA Magnetic Isolation Module (NEB, E7490). The enriched and purified mRNA was broken into approximately 200 nt short RNA inserts, which were used to synthesize the first-strand cDNA and the second cDNA. The double-stranded cDNA were performed end-repair/dA-tail and adaptor ligation. The suitable fragments were isolated by AgencourtAMPure XP beads (Beckman Coulter, Inc.), and enriched by PCR amplification. Finally, the constructed cDNA libraries of the samples were sequenced on a flow cell using an Illumina HiSeq™ 2500 sequencing platform.

Transcriptome analysis was done using reference genome-based reads mapping. Low quality reads, such as adaptor sequences, unknown nucleotides>5%, or Q20 <20% (percentage of sequences with sequencing error rates <1%), were removed by perl script. The clean reads, which were filtered from the raw reads, were mapped to Nipponbare (IRGSP-1.0 pseudomolecule/MSU7) reference genome using Bowtie2 and Tophat2 software^37^. The aligned records from the aligners in BAM/SAM format were further examined to remove potential duplicate molecules. Gene expression levels were estimated using FPKM values (Fragments Per Kilobase of transcript per Million fragments mapped) by the Cufflinks software^38^.

DESeq was employed to evaluate the differential gene expression between F_1_ and its parents. After that, gene abundance differences between those samples were calculated based on the ratio of the FPKM values. The false discovery rate (FDR) control method was used to identify the threshold of the *P*-value in multiple tests in order to compute the significance of the differences. Here, only genes with an absolute value of Fold Change c and FDR significance score <0.01 were used for subsequent analysis.

GO analysis was performed for the functional categorization of differentially expressed genes using David analysis tools (http://david.abcc.ncifcrf.gov/home.jsp), the Plant GeneSet Enrichment Analysis Toolkit (http://structuralbiology.cau.edu.cn/PlantGSEA/) and AgriGO tool (http://bioinfo.cau.edu.cn/agriGO/). Cluster analysis was performed using Cluster 3.0 software. Pathway analysis was performed using PlantGSEA software. Predicted protein-protein interactions were analyzed using STRING software (http://www.string-db.org/). Annotations for differentially expressed genes were retrieved from the Rice Genome Annotation Project (http://rice.plantbiology.msu.edu/). Gene co-expression analysis was performed by using Gene Co-expression Networks in the website (http://bioinfo.sibs.ac.cn/carmo/Gene_Annotation.php). Venny software was used to identify the overlapped differentially expressed genes in different samples and tissues (http://bioinfogp.cnb.csic.es/tools/venny/)^10^.

### miRNA analysis

Anthers at meiosis stage were collected from Huaduo 3, Huajingxian 74-4x and their hybrid. Total RNA was isolated from the anthers by using Trizol reagent (Invitrogen, CA, USA) according to the manufacturer’s protocol. The quantity and purity of total RNA were analyzed by RNA 6000 Nano LabChip Kit (Agilent, CA, USA) and Bioanalyzer 2100 with RIN number >7.0. Small RNA library was prepared by using about 1 μg of RNA according to the protocol of Illumina’s TruSeq small RNA sample preparation Kits (San Diego, USA). Single-end sequencing (36 bp) was executed on an Illumina Hiseq2500 at the LC-BIO (Hangzhou, China) following the manufacturer’s protocol. After sequencing, the data was further analyzed with an in-house program, ACGT101-miR, to remove common RNA families (snRNA, snoRNA, rRNA and tRNA), low complexity, adapter dimers, junk and repeats. Then, the unique sequences with 18–25 nt length were BLASTed to rice precursors in miRBase 20.0 (ftp://mirbase.org/pub/mirbase/CURRENT/) to detect known miRNAs. RNAfold software (http://rna.tbi.univie.ac.at/cgi-bin/RNAfold.cgi) was employed to identify the novel predicted miRNAs. The miRNAs were considered as differentially expressed according to the normalized deep-sequencing levels (with the exclusion of 10 RPM) in F_1_ and its two parents. Chi-square (*X*^*2*^) test and Fisher exact test were used to estimate *P-value*. miRNAs with *P-value* < 0.05 and log_2_ (fold change ratio) >1 were considered as differentially expressed miRNAs. Targeted genes of differentially expressed miRNAs were predicted by using psRNATarget (http://plantgrn.noble.org/psRNATarget/)^22^,^39^.

Whole-genome re-sequencing, transcriptome and small RNA sequencing data have been deposited to NCBI SRA database (SRA accession: SRP078960).

### qRT-PCR

A set of 12 selected DEGs from the transcriptome analysis were validated by qRT-PCR using the same RNA samples that used for transcriptome analysis. The sequences of corresponding target genes were obtained from the National Center for Biotechnology Information (NCBI). The target gene sequences were used to design the 12 gene-specific primer pairs using Primer Premier5.0 and Oligo6.0 software (Supplementary Table S14). About 1 μg of isolated RNA was used to synthesize first-strand complementary DNA (cDNA) using the Transcriptor First Strand cDNA Synthesis Kit (Roche). qRT-PCR was performed using SYBR Green (Bio-Rad) in a LightCycler480 System (Roche). The final reaction volume for qRT-PCR was 20 μL, and each reaction contained 2 μL of cDNA, 10 μM of forward and reverse primers, and 10 μL of universal SYBR Green supermix (Bio-RAD). The following amplification program was used: denaturation at 95 °C for 30 s and 40 cycles of amplification (95 °C for 5 s followed by 58 °C for 20 s). Melting curve analysis was performed from 65 °C to 95 °C, with 5-s increments of 0.5 °C. The rice Ubiquitin gene was used as an internal control. The relative expression levels were calculated as 2^−(ΔCt of treatment−ΔCt)^ of control^40^, where Ct represents threshold cycle. Each PCR reaction, including the control reaction, was performed in triplicate^10^.

## Additional Information

**How to cite this article**: Guo, H. *et al*. Transcriptome analysis of neo-tetraploid rice reveals specific differential gene expressions associated with fertility and heterosis. *Sci. Rep.* **7**, 40139; doi: 10.1038/srep40139 (2017).

**Publisher's note:** Springer Nature remains neutral with regard to jurisdictional claims in published maps and institutional affiliations.

## Supplementary Material

Supplementary Information

Supplementary Dataset 1

Supplementary Dataset 2

Supplementary Dataset 3

Supplementary Dataset 4

## Figures and Tables

**Table 1:** Heterosis analysis of the F_1_ hybrid (Huajingxian 74-4x ×Huaduo 3) and its parents.

Traits	H3	T452	F_1_ hybrid	HPH (%)	MPH (%)
PH (cm)	121.67±3.51	92.00±1.73	126.67±0.58	4.11	18.56
EP	4.00±0.32	4.33±2.08	8.33±2.52	92.31	100.00
FG	123.25±16.88	107.91±19.43	131.78±11.16	6.92	14.02
SS (%)	83.93±6.41	11.84±0.48	87.86±5.09	4.69	83.49
GYP (g)	16.03±0.23	1.64±0.51	37.07±16.67	131.27	319.70
GL (cm)	8.90±0.32	10.00±0.35	9.50±0.01	−5.00	0.53
GW (cm)	4.00±0.36	3.10±0.21	3.13±0.15	−21.67	−11.74

H3: Huaduo 3; T452: Huajingxian 74-4x; F_1_ hybrid: (Huajingxian 74-4x ×Huaduo 3); HPH: High-parent heterosis; MPH: Mid-parent heterosis.

PH: Plant height; EP: Effective number of panicles per plant; FG: Filled grains per panicle; SS: Seed setting; GYP: Grain yield per plant; GL: Grain length (10-grains); GW: Grain width (10-grains).

**Table 2:** Differentially expressed genes (DEGs) associated with anther, ovary and leaf.

Name of combination	Total DEGs			Up-DEGs			Down-DEGs		
	Total No	Known DEG	New gene	No of Up DEG	Known DEG	New gene	No of down DEG	Known DEG	New gene
H3vsT452-AN*	2189	1273	916	1318	826	492	871	447	424
F_1_vsT452- AN	2498	1405	1093	1781	991	790	717	414	303
F_1_vsH3- AN	1693	1131	562	1068	594	474	625	537	88
H3vsT452-OV	2286	1319	967	1349	835	514	937	484	453
F_1_vsT452- OV	2424	1354	1070	1674	942	732	750	412	338
F_1_vsH3- OV	1521	973	548	1030	569	461	491	404	87
H3vsT452-LE	2166	1824	342	840	693	147	1326	1131	195
F_1_vsT452-LE	1543	1362	181	1012	851	161	531	511	20
F_1_vsH3-LE	1557	1389	168	725	598	127	832	791	41
Total	17877	12030	5847	10797	6899	3898	7080	5131	1949

^*^H3: Huaduo 3; T452: Huajingxian 74-4x; F_1_: (Huajingxian 74-4x × Huaduo 3); AN: Anther; OV: Ovary; LE: Leaf.

**Table 3:** Identification of differentially expressed miRNAs (DERs) in anther.

Name of combination	Total DER		Up-DER		Down-DER	
	Total No	Novel miRNA	No of Up DER	Novel miRNA	No of down DER	Novel miRNA
H3vsT452-AN*	122	35	82	33	40	2
F_1_vsT452-AN	83	21	34	9	49	12
F_1_vsH3- AN	93	20	50	19	43	1
Total	298	76	166	61	132	15
DERFu- AN	38	13	24	12	14	1

^*^H3: Huaduo 3; T452: Huajingxian 74-4x; F_1_: (Huajingxian 74-4x × Huaduo 3); AN: Anther.

**Table 4:** Nonadditive differentially expressed genes (NDEGs) and number of differentially expressed genes uniquely associated with F_1_ and specific to (DEGFu-sp) anther, ovary, and leaf in F_1_ hybrid.

Tissues	No. of DEGFu		No. of NDEGs		Total	%*
	Total	Specific	Up	Down		
Anther	1150	807	197	117	314	0.09
Ovary	1014	663	386	192	578	1.61
Leaf	1122	866	312	20	332	1.5
Total	3286	2336	895	329	1224	3.2

^*^Indicate the percent ratio of NDEGs to total expressed genes in F_1_.
