# Supplementary material for: Transcriptome analysis of neo-tetraploid rice reveals specific differential gene expressions associated with fertility and heterosis
Source: Sci Rep. 2017 Jan 10;7:40139. doi: 10.1038/srep40139 (PMC5223177; doi:10.1038/srep40139)
Supplement: Supplementary Information [file srep40139-s1.pdf]

Supplementary information

Transcriptome analysis of Neo-tetraploid rice reveals specific differential gene expressions associated with fertility and heterosis

Haibin Guo†, Mendrikahy Jean Nestor†, Lei Xie†, Junfeng Deng, Zijun Lu, Jinwen Wu, Xiang Li, Muhammad Qasim Shahid*, Xiangdong Liu*

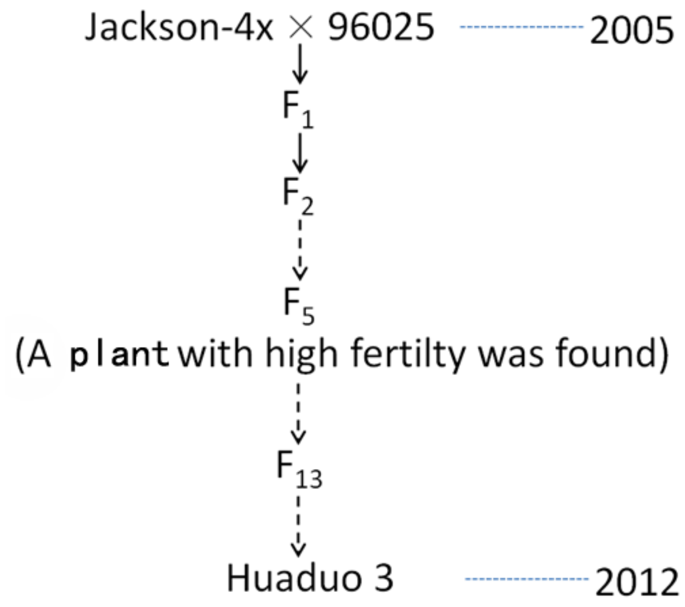

Fig. S1. Breeding process of Huaduo 3

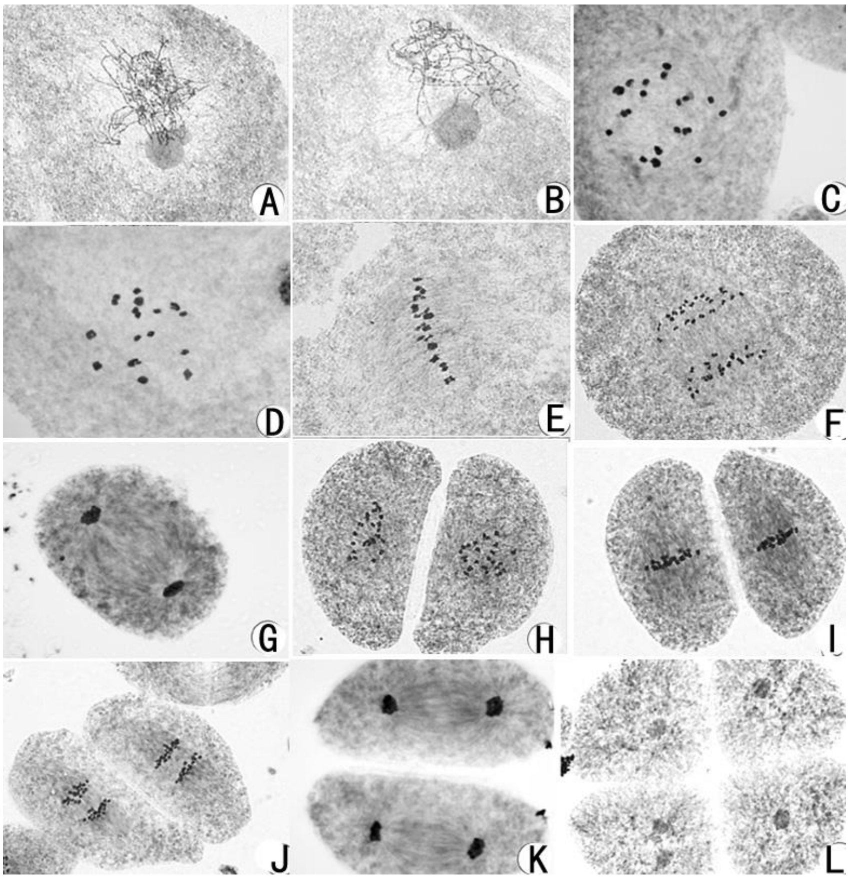

Fig. S2. Chromosome behavior during pollen mother cell meiosis in Huaduo 3.
(A) zygotene; (B) pachytene; (C, D) diakinesis; (E) metaphase Ⅰ; (F) anaphase Ⅰ; (G) telophase Ⅰ; (H) prophase Ⅱ; (I) metaphase Ⅱ; (J) anaphase Ⅱ; (K) telophase Ⅱ; (L) the tetrad stage.

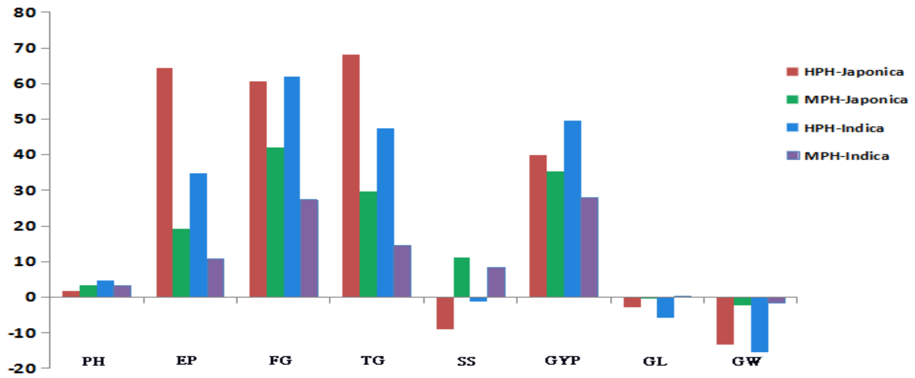

Fig. S3. Heterosis analysis of the hybrids generated by crossing Huaduo 3 (H3) with other autotetraploid rice lines.

HPH-*japonica*: High-parent heterosis of H3 and *japonica*; MPH-*japonica*: Mid-parent heterosis of H3 and *japonica*; HPH-*indica*: High-parent heterosis of H3 and *indica*; MPH-*indica*: Mid-parent heterosis of H3 and *indica*

PH: Plant height; EP: Effective number of panicles per plant; FG: Filled grains per plant; TG: Total grains per plant; SS: Seed setting; GYP: Grain yield per plant; GL: Grain length (10-Grains); GW: Grain width (10-Grains)

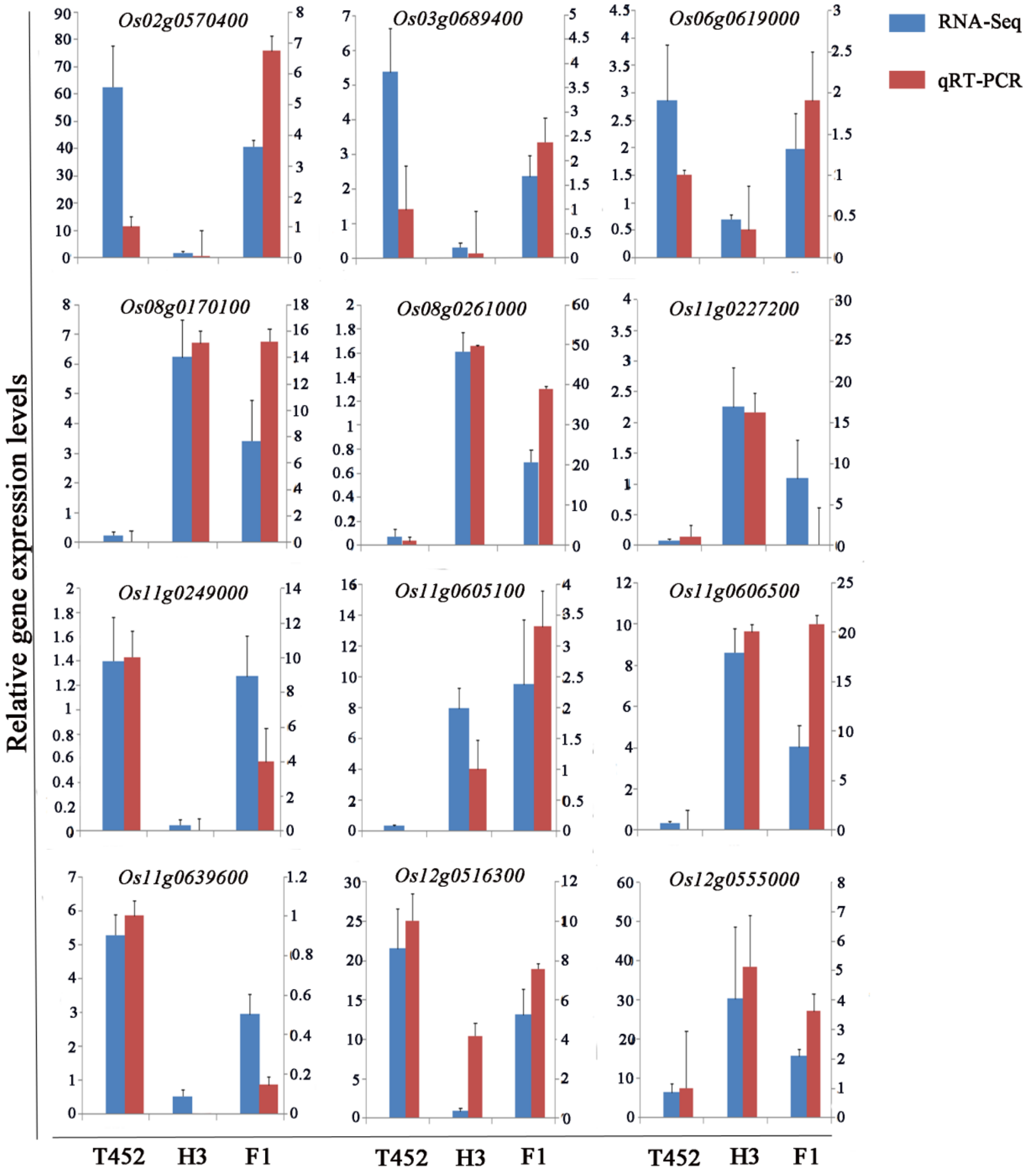

Fig. S4. qRT-PCR validation for the quality of transcriptomic data.

The blue columns represent the expression level of the transcript (y-axis), and the red columns represent the relative gene expression levels by using qRT-PCR from three independent replicates (y-axis).

H3: Huaduo 3; T452: Huajingxian 74-4x; F_1: (Huajingxian 74-4x × Huaduo 3)

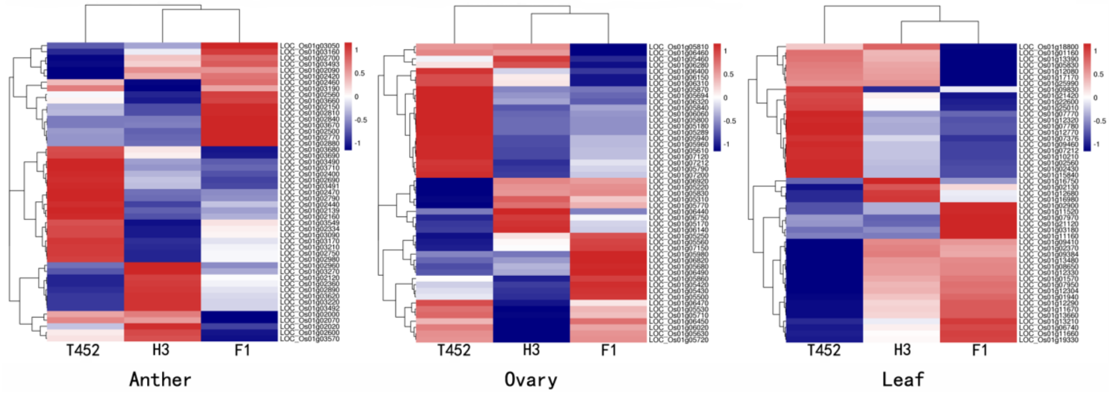

Fig. S5. Clustering of differential gene expressions (DGEs) of anther, ovary and leaf in F_1 compared to its parents.

H3: Huaduo 3; T452: Huajingxian 74-4x; F_1: (Huajingxian 74-4x × Huaduo 3)

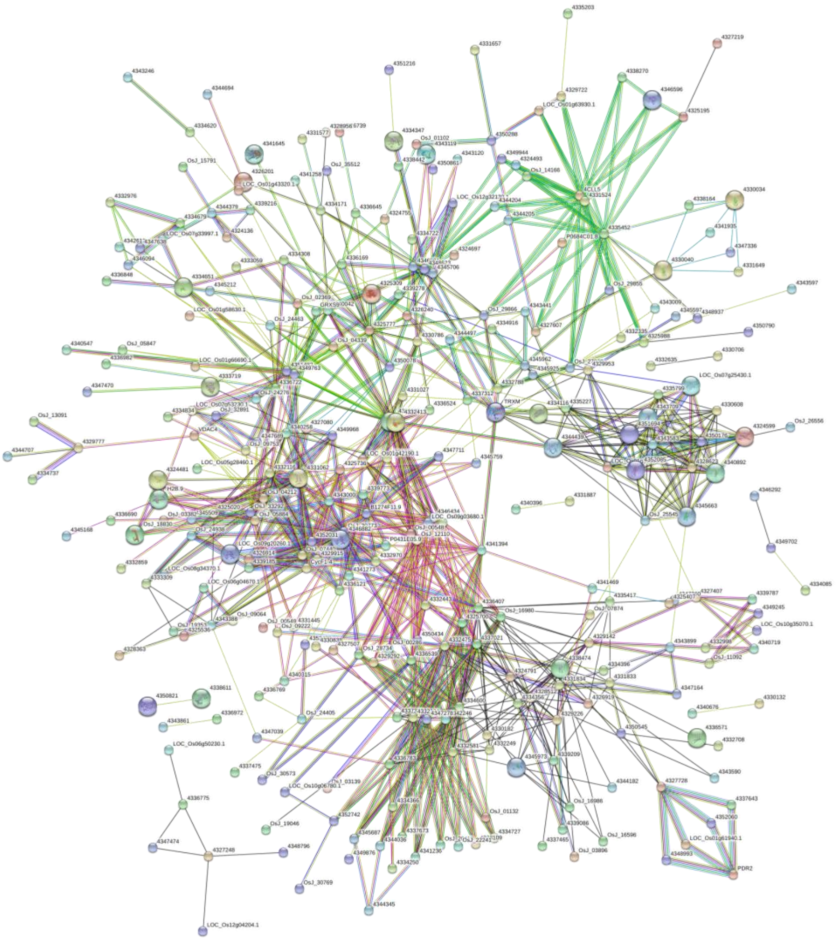

Fig. S6a. Predicted protein-protein interaction network of differentially expressed genes uniquely belonging to F_1 compared to its parents (DEGFu-sp) in anther.

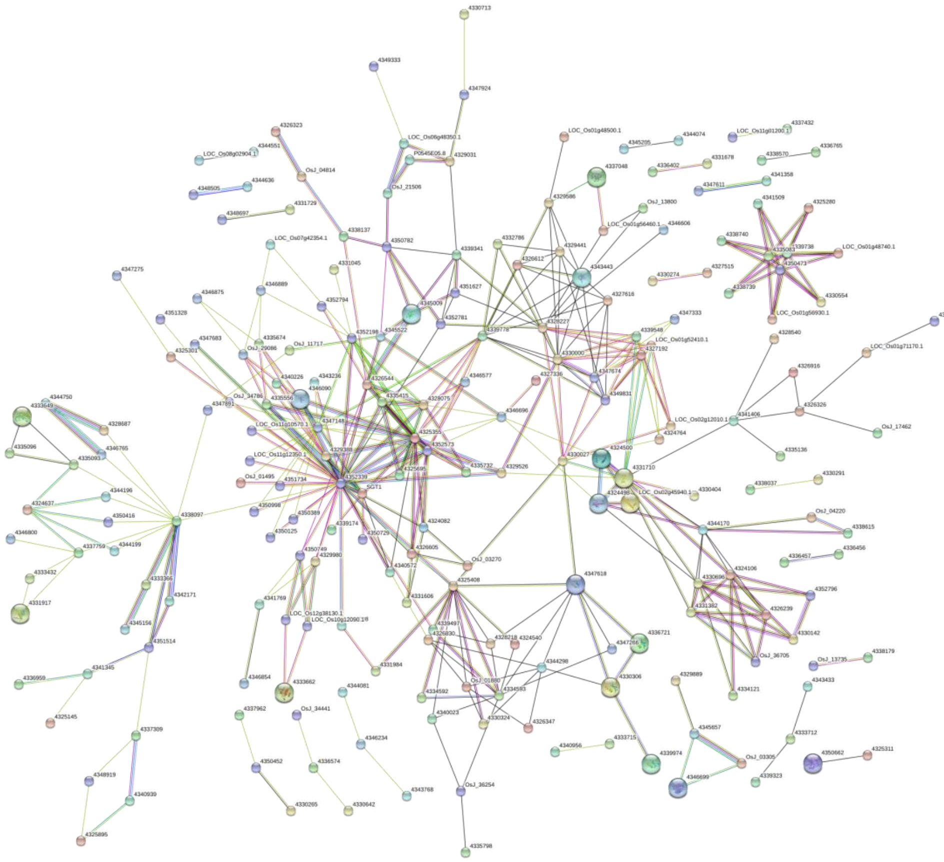

Fig. S6b. Predicted protein-protein interaction network of differentially expressed genes uniquely belonging to F_1 compared to its parents (DEGFu-sp) in ovary.

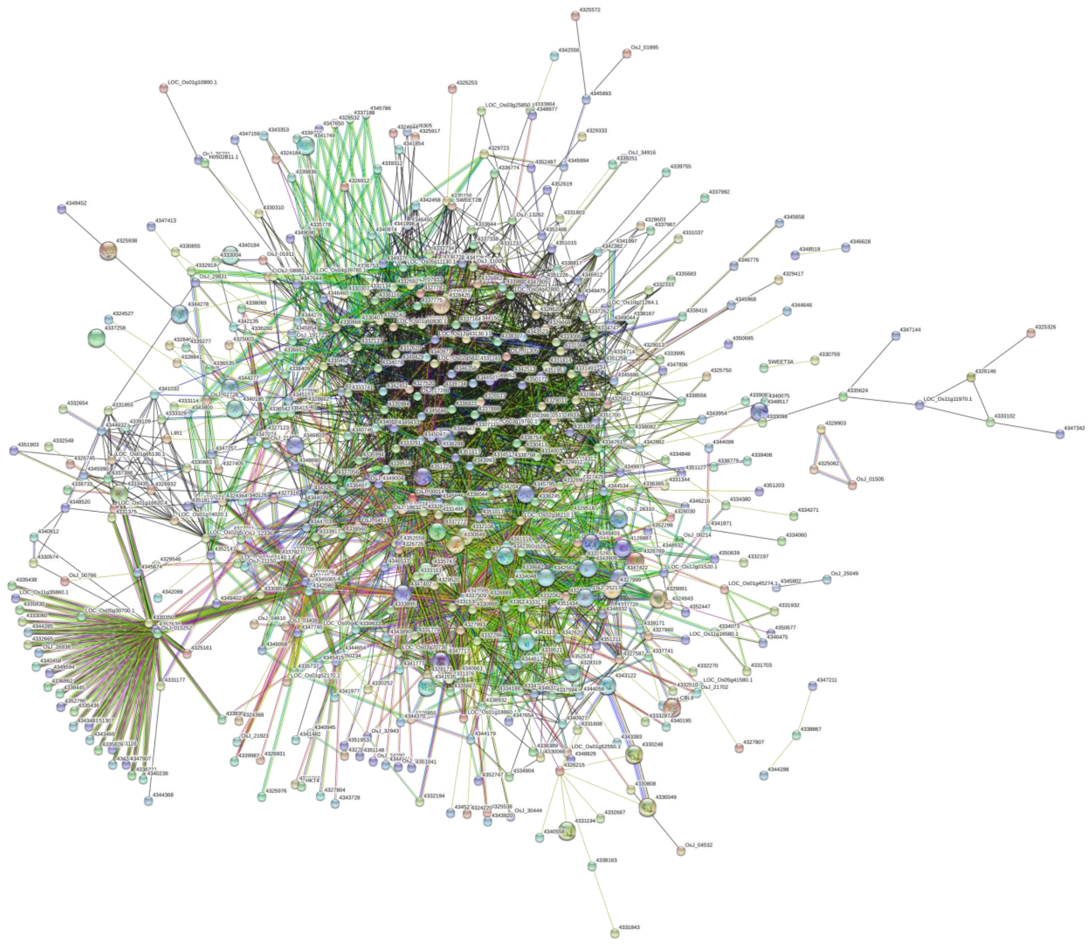

Fig. S6c. Predicted protein-protein interaction network of differentially expressed genes uniquely belonging to F_1 compared to its parents (DEGFu-sp) in leaf.

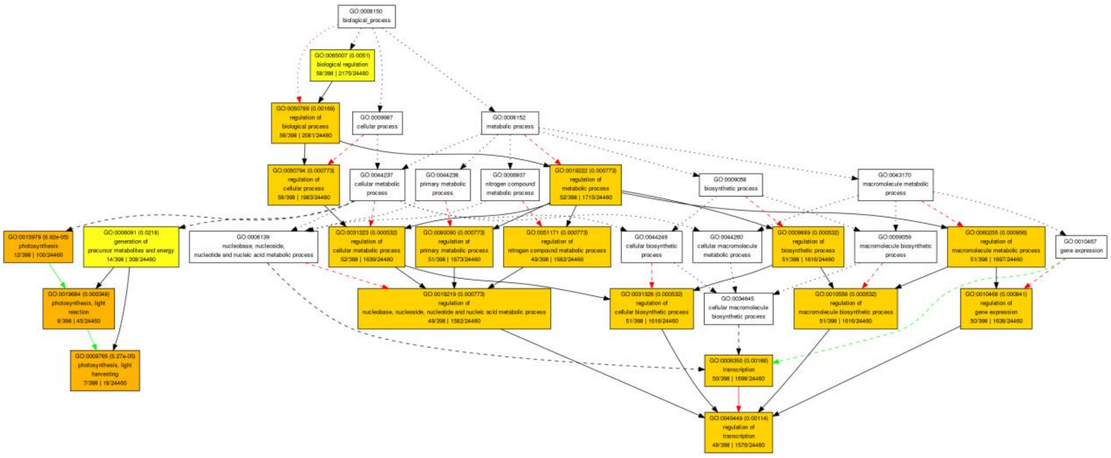

Fig. S7. GO analysis of differentially expressed genes uniquely belonging to F_1 compared to its parents (DEGFu-sp) of the Biological process category in anther.

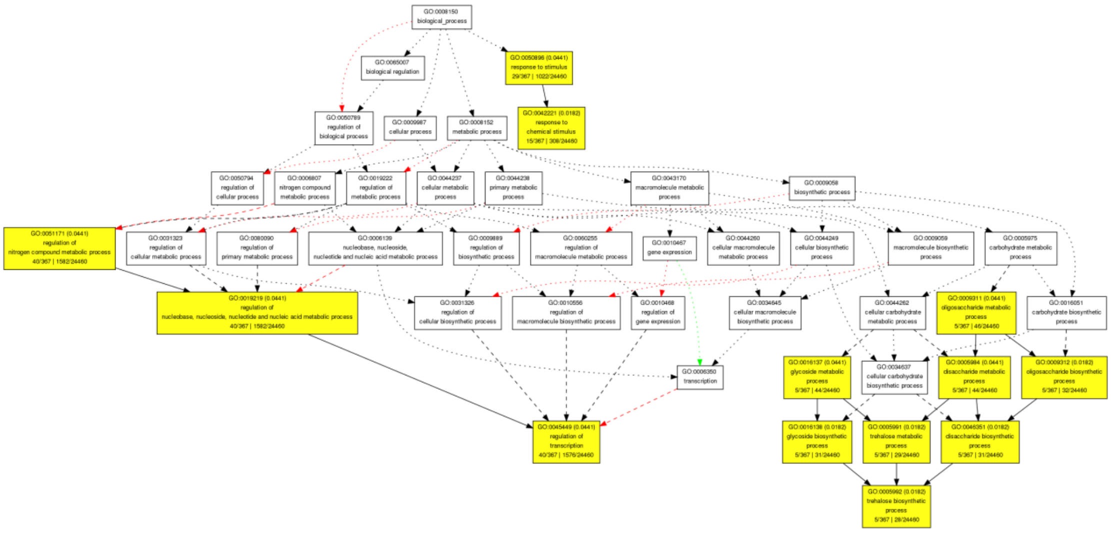

Fig. S8a. GO analysis of differentially expressed genes uniquely belonging to F_1 compared to its parents (DEGFu-sp) of the Biological process category in ovary.

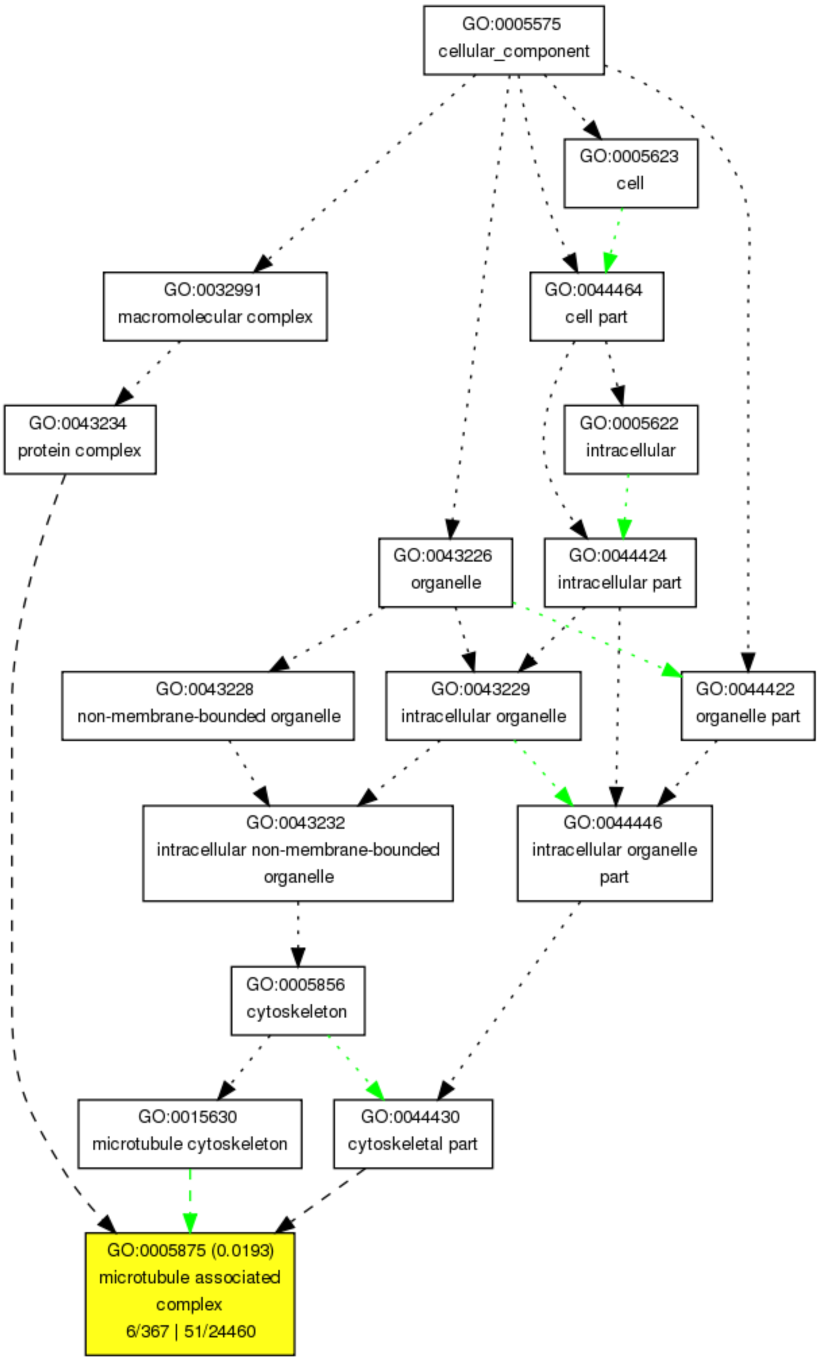

Fig. S8b. GO analysis of differentially expressed genes uniquely belonging to F_1 compared to parents (DEGFu-sp) of the cellular component category in ovary.

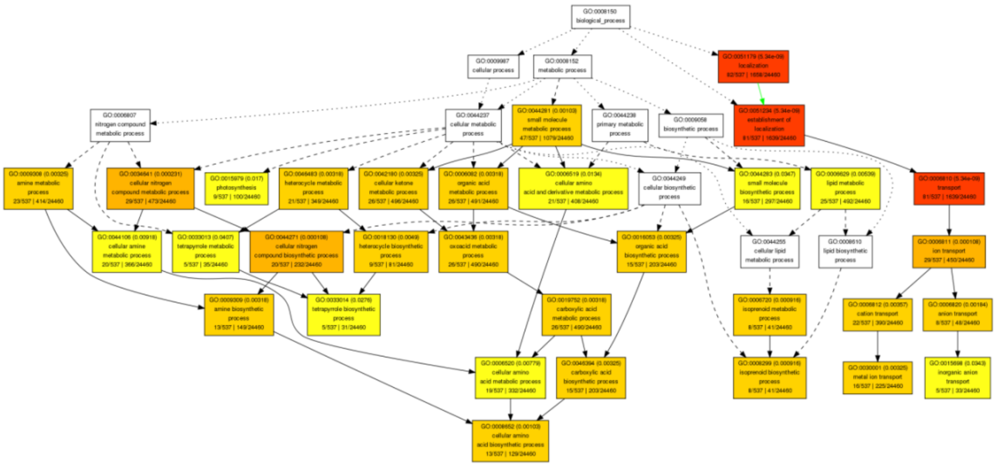

Fig. S9. GO analysis of differentially expressed genes uniquely belonging to F_1 compared to parents (DEGFu-sp) of the Biological process category in leaf.

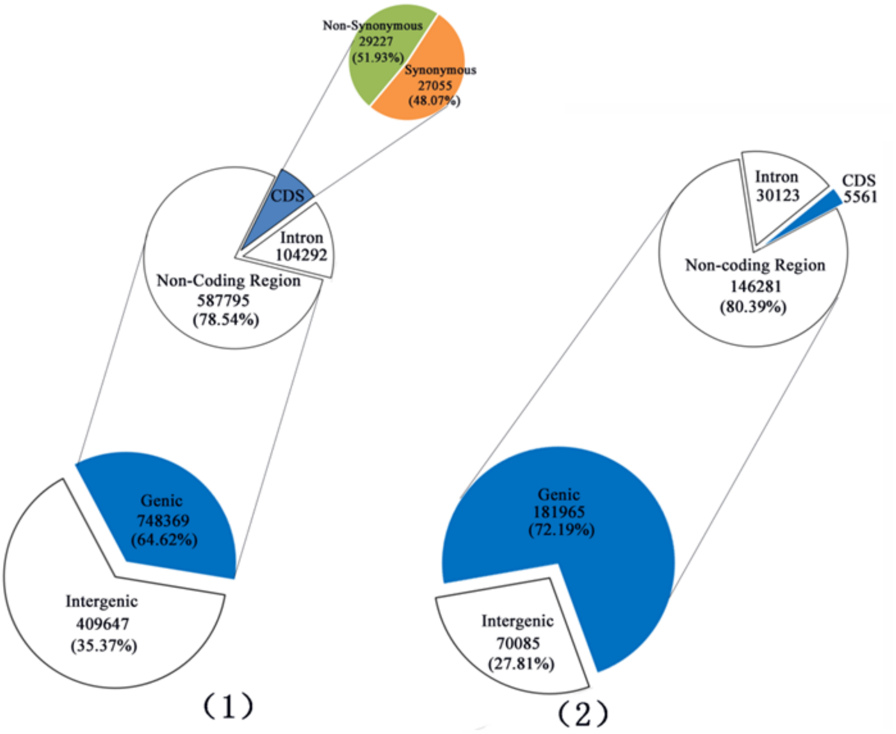

Fig. S10. SNP and Indel variations between Huajingxian 74-4x (T452) and Huaduo 3 (H3).

(1) SNP; (2) Indel; CDS (coding sequences)

Table S1. Agronomic traits of Huaduo 3, autotetraploid rice parents and their hybrids.

Name	PH (cm)	EP	FG	TG	SS (%)	GYP(g)	GL(cm)	GW(cm)
Indica lines	110.95	4.95	218.85	500.47	41.38	6.65	10.17	3.33
Japonica lines	104.94	3.94	92.64	277.04	25.97	2.83	8.99	3.79
Huaduo 3	121.67	4.00	412.00	493.00	83.93	16.03	8.90	4.00
JI	128.40	6.23	351.20	635.12	54.61	12.59	9.73	3.60
HIH	131.65	6.51	686.46	813.54	83.07	24.49	9.54	3.40
HJH	128.25	6.92	661.73	828.84	76.45	22.42	8.92	3.52

JI indicates hybrids developed by crossing *japonica* with *indica* autotetraploid rice lines.

HIH indicates hybrids developed by crossing Huaduo 3 with *indica* autotetraploid rice lines.

HJH indicates hybrids developed by crossing Huaduo 3 with *japonica* autotetraploid rice lines.

PH: Plant height; EP: Effective number of panicles per plant; FG: Filled grains per plant; TG: Total grains per plant; SS: Seed setting; GYP: Grain yield per Plant; GL: Grain length (10-grains); GW: Grain width (10-grains)

Table S2. Heterosis analysis of hybrids generated by the crossing of Huaduo 3 and *indica* autotetraploid rice lines.

Main traits	High parent heterosis					Mid parent heterosis				
	Average value	Variable coefficient	Variation range	+	-	Average value	Variable coefficient	Variation range	+	-
PH	4.71	1.74	-12.88~27.82	19	7	3.24	0.83	-0.43~10.18	25	1
EP	34.90	1.35	-66.67~137.50	22	4	10.66	1.23	-11.84~38.41	23	3
FG	61.95	1.18	-65.96~240.53	21	5	27.30	0.89	-9.76~91.77	25	1
TG	47.38	1.41	-53.19~212.07	20	4	14.51	1.14	-8.79~54.60	24	2
SS	-1.33	-7.83	-44.10~9.08	15	11	8.40	0.68	-0.41~21.11	25	1
GYP	49.51	1.31	-55.65~216.41	20	6	27.87	0.93	-4.34~94.45	25	1
GL	-5.80	-1.37	-22.05~6.62	7	19	0.02	72.63	-2.70~2.50	15	11
GW	-15.35	-0.35	-24.38~-3.19	0	26	-1.70	-0.79	-5.05~0.19	1	25

Table S3. Heterosis analysis of hybrids generated by the crossing of Huaduo 3 and *japonica* autotetraploid rice lines.

	High parent heterosis					Mid parent heterosis				
Main traits	Average value	Variable coefficient	Variation range	+	-	Average value	Variable coefficient	Variation range	+	-
PH	1.84	8.09	-34.11~32.99	8	6	3.29	1.18	-6.81~9.33	12	2
EP	64.46	0.76	0~182.35	14	0	19.10	0.69	4.43-47.73	14	0
FG	60.61	0.86	-3.40~13.32	13	1	42.03	0.56	20.49~92.80	14	0
TG	68.12	0.68	10.75~136.87	14	0	29.75	0.59	8.15~66.04	14	0
SS	-8.91	-1.76	-50.50~8.89	5	9	11.07	0.75	-1.67~22.18	11	3
GYP	39.88	1.39	-23.65~142.00	10	4	35.31	0.65	3.89~82.82	14	0
GL	-2.77	-3.47	-33.99~8.99	5	9	-0.08	-31.75	-8.47~2.29	8	6
GW	-13.25	-0.77	-35.95~-1.54	0	14	-2.24	-0.99	-8.43~-0.58	0	14

PH: Plant height; EP: Effective number of panicles per plant; FG: Filled grains per plant; TG: Total grains per plant; SS: Seed setting; GYP: Grain yield per plant; GL: Grain length (10-grains); GW: Grain width (10-grains)

+: the number of hybrids with positive heterosis; - : the number of hybrids with negative heterosis

Supplementary Table S4. The list of differentially expressed genes that uniquely associated with F_1 compared to parents and specific (DEGFu-sp) to anther, ovary and leaf.

(Please see the supplementary Excel file).

Supplementary Table S5. Differentially expressed genes uniquely belonging to F_1 compared to T452 (DEGFu-sp) in anther

(Please see the supplementary Excel file).

Supplementary Table S6. Identification of miRNAs associated with DEGFu-sp to anther and their target genes.
(Please see the supplementary Excel file).

Supplementary Table S7. DEGFu-nonadditive genes in anther, ovary and leaf
(Please see the supplementary Excel file).

Table S8. SNPs and Indels mapped to reference genome and specific to two parents.

Name	SNPs	Specific SNPs	Indels	Specific Indels
Huajingxian 74-4x	1976579	1192412	415252	248194
Huaduo 3	1158627	374460	251254	84196
Total	2351039	1566872	499448	332390

Table S9. SNP and Indel variations between Huajingxian 74-4x (T452) and Huaduo 3 (H3).

Type	Total	Non-polymorphic loci	Polymorphic loci	Polymorphism rate (%)
SNP	1704411	566368	1138043	66.77
Indel	360400	108227	252173	69.97

Table S10. Different loci statistics of SNPs and Indels variations between Huajingxian 74-4x and Huaduo 3.

Class	SNPs		Class	Indels	
	No	Rate (%)		No	Rate (%)
INTERGENIC	409647	35.3717	INTERGENIC	70085	27.7843
UPSTREAM	311044	26.8577	UPSTREAM	75667	29.9972
DOWNSTREAM	241003	20.8098	DOWNSTREAM	58947	23.3688
UTR_5_PRIME	9401	0.8117	UTR_5_PRIME	4622	1.8323
UTR_3_PRIME	21372	1.8454	UTR_3_PRIME	6344	2.515
INTRAGENIC	104	0.009	INTRAGENIC	197	0.0781
INTRON	104292	9.0053	INTRON	30123	11.9419
SPLICE_SITE_ACCEPTOR	86	0.0074	SPLICE_SITE_ACCEPTOR	55	0.0218
SPLICE_SITE_DONOR	105	0.0091	SPLICE_SITE_DONOR	82	0.0325
SPLICE_SITE_REGION	2158	0.1863	SPLICE_SITE_REGION	564	0.2236
START_GAINED	1984	0.1713	FRAME_SHIFT	2001	0.7933
START_LOST	89	0.0077	START_LOST	37	0.0147
STOP_GAINED	405	0.035	STOP_GAINED	41	0.0163
STOP_LOST	148	0.0128	STOP_LOST	42	0.0167
SYNONYMOUS_CODING	27006	2.3319	CODON_CHANGE(INSERTION)	337	0.1336
NON_SYNONYMOUS_CODING	29219	2.523	CODON_CHANGE(DELETION)	611	0.2422
SYNONYMOUS_STOP	49	0.0042	CODON_INSERTION	1372	0.5439
NON_SYNONYMOUS_START	8	0.0007	CODON_DELETION	1120	0.444

Table S11. Names and the types of parents used to prepare autotetraploid rice hybrids.

Code No.	*Indica* autotetraploid parents		Code No.	*Japonica* autotetraploid parents	
	Name	Source		Name	Source
T41	Aijiaonante-4x	Lab*	T47	Liaojing 944-4x	Lab
T44	96025-4x	ICS,CAAS**	T48	Yanjing 48-4x	Lab
T412	Dadanbai86-4x	Lab	T413	J455-4x	Lab
T416	Yuhei 1-4x	Lab	T415	Nanhaizhaoyinzhan-4x	Lab
T417	Xichuan-4x	Lab	T422	J422-4x	Lab
T419	Yuexiangzhan-4x	Lab	T424	Shennong 15-4x	Lab
T420	Dayebai-4x	Lab	T431	Taichung 65-4x	Lab
T421	Guinongzhan-4x	Lab	T432	E2-4x	Lab
T428	Linglun-4x	Lab	T433	E4-4x	Lab
T442-1	4001-1-4x	Lab	T434	E5-4x	Lab
T443	CypressPB-6-4x	SCBG,CAS***	T435	E24-4x	Lab
T444	Gulfmont-4x	SCBG,CAS	T437	E45-4x	Lab
T445	Luxiang-97-4x	SCBG,CAS	T438	E245-4x	Lab
T453	PII-6-4x	Lab	T450	02428-4x	Lab
T447	IR36-4x	Lab			
T448	8821-4x	Lab			
T451	Dalinuo-4x	Lab			
T452	Huajingxian 74-4x	Lab			
T456	M12-4x	Lab			
T461	Wuxienuo-4x	ICS,CAAS			
T463	Nanjing 11-4x	ICS,CAAS			
T465	Nantehao-4x	ICS,CAAS			
T467	Peiai64-4x	ICS,CAAS			
T469	Huayinzhan-4x	Lab			
T473	Huahui305-4x	Lab			
T479	N39s-4x	Lab			

Lab*: State Key Laboratory for Conservation and Utilization of Subtropical Agro-bioresources, South China Agricultural University

ICS,CAAS**: Institute of Crop Sciences, Chinese Academy of Agricultural Sciences

SCBG,CAS***: South China Botanical Garden, Chinese Academy of Sciences

Table S12. Autotetraploid rice hybrids generated in the present study.

No.	Crossing parents	No.	Crossing parents	No.	Crossing parents
1	T437×T41*	12	T435×T413	23	T433×T453
2	T424×T419	13	T438×T413	24	T434×T453
3	T424×T420	14	T432×T416	25	T435×T453
4	T424×T442	15	T433×T416	26	T437×T453
5	T424×T444	16	T434×T416	27	T438×T453
6	T424×T445	17	T435×T416	28	T434×T455
7	T424×T447	18	T433×T443	29	T435×T455
8	T424×T448	19	T434×T443	30	T437×T455
9	T424×T451	20	T431×T452	31	T434×T456
10	T424× T463	21	T432×T452	32	T434×T456
11	T434×T413	22	T434×T452	33	T433×T469

* : See Table S11 for the names of autotetraploid rice lines

Table S13. Hybrids developed by crossing Huaduo 3 with different types of autotetraploid rice lines

No.	HIH*	No.	HIH	No.	HJH**
1	H3***×T41****	15	H3×T447	1	H3 xT47
2	H3×T44	16	H3×T448	2	H3 xT48
3	H3×T412	17	H3×T451	3	H3×T413
4	H3×T416	18	H3×T452	4	H3×T415
5	H3×T417	19	H3×T456	5	H3×T422
6	H3×T419	20	H3×T461	6	H3×T424
7	H3×T420	21	H3×T463	7	T431 ×H3
8	H3×T421	22	H3×T465	8	T432×H3
9	T428×H3	23	H3×T467	9	T433 ×H3
10	H3×T442-1	24	H3×T469	10	T434 ×H3
11	H3×T443	25	H3×T473	11	T435 ×H3
12	H3×T444	26	T479 ×H3	12	T437 ×H3
13	H3×T445			13	T438 ×H3
14	H3×T453			14	H3×T450

* : HIH indicates hybrids developed by crossing Huaduo 3 with *indica* autotetraploid rice lines.

** : HJH indicates hybrids developed by crossing Huaduo 3 with *japonica* autotetraploid rice lines.

*** : H3 indicates Huaduo 3. **** : See Table 11 for the names of autotetraploid rice lines.

Table S14. List of primers used for qRT-PCR

No.	Gene Symbol	Primer(pair)	Amplicon length (bp)
1	*LOC_Os02g36140*	CAACAGACTCGACCAGCTACATT<br>CGATAACAGCCGTCAACCAA	140
2	*LOC_Os03g48320*	CGTGAGCCTTGAATCCGTAG<br>GCATCCAAGTCCTCCCAAAC	186
3	*LOC_Os06g41480*	CTTGAGAATTGCATGGGTTGG<br>GCCCTTCATTTCACCGTTCC	135
4	*LOC_Os08g07330*	AGCTCATCAACGGAATCAAGG<br>AGAGTCATCACGAGAAAGTGGC	212
5	*LOC_Os08g16120*	GTTGCTGTTTGTGGTATGGGTG<br>TCGTAGTTTACTTCGCTGCTGTC	182
6	*LOC_Os11g12000*	GAGTATGAGCGGGAGAAGAACA<br>CAAGTCACGAGGGTCCAAATC	177
7	*LOC_Os11g14380*	TTGATTCCAAGGATGCGAAGG<br>CGAACACTAGGCCCAGATGC	157
8	*LOC_Os11g39190*	ACGGTACATCAATAGTCCCAACG<br>TTCTGCCTGAACAACATCCTCT	193
9	*LOC_Os11g39320*	AGCAGCAATGGGAAGGTGA<br>CAAAGATCGGACATGCGACA	216
10	*LOC_Os11g42040*	TTGTATTTGACGATGTATGGGTGC<br>TCCAGCGAACTAGATGGTGTTT	172
11	*LOC_Os12g33160*	GCAATGAACACCCTCCTCCC<br>TGGCATCCTCGATGTCGTAA	220
12	*LOC_Os12g36830*	GCCATGCCCAAGGTTTGT<br>CCATCATCCACAGCAGGGTT	107
13	*Ubiquitin*	CAAGATGATCTGCCGCAAATGC<br>TTTAACCAGTCCATGAACCCG	103
